# Risk Factors of Delirium in Sequential Sedation Patients in Intensive Care Units

**DOI:** 10.1155/2017/3539872

**Published:** 2017-10-31

**Authors:** Jie Yang, Yongfang Zhou, Yan Kang, Binbin Xu, Peng Wang, Yinxia Lv, Zhen Wang

**Affiliations:** Department of Critical Care Medicine, West China Hospital of Sichuan University, Chengdu 610041, China

## Abstract

**Background:**

Delirium is a primary adverse event in ventilated patients who receive long-term monosedative treatment. Sequential sedation may reduce these adverse effects. This study evaluated risk factors for delirium in sequential sedation patients.

**Methods:**

A total of 141 patients who underwent sequential sedation were enrolled. Delirium was diagnosed using Confusion Assessment Method for the Intensive Care Unit (CAM-ICU) scale. Univariate and multivariate Cox proportional hazards regressions were used to predict risk factors.

**Results:**

Older age (≥51) (RR = 2.432, 95% CL 1.316–4.494, *p* = 0.005), higher SOFA score (≥14) (RR = 2.022, 95% CL 1.076–3.798, *p* = 0.029), regular smoking (RR = 2.366, 95% CL 1.277–4.382, *p* = 0.006), and higher maintenance dose of midazolam (RR = 1.052, 95% CL 1.000–1.107, *p* = 0.049) and fentanyl (RR = 1.045, 95% CL 1.019–1.072, *p* = 0.001) when patients met sequential criteria, were independent risk factors of delirium. Sequential sedation with dexmedetomidine (RR = 0.448, 95% CL 0.209–0.963, *p* = 0.040) was associated with a lower risk of delirium.

**Conclusions:**

Older age, higher SOFA score, regular smoking, and higher maintenance dose of midazolam and fentanyl when patients met sequential criteria were independent risk factors of delirium in sequential sedation patients. Sequential sedation with dexmedetomidine reduced risk of delirium.

## 1. Introduction

Delirium is a disorder of patient consciousness that is characterized by four aspects: an acute change in patient mental status, loss of attention, disturbance in thinking, and cognitive dysfunction [1]. Delirium results from various causes in intensive care unit (ICU) patients.

Previous studies demonstrated that patients under mechanical ventilation exhibit a higher risk of experiencing delirium than nonmechanically ventilated patients (20%–50%) [2]. The occurrence of delirium is also more frequent in elderly patients [3–5], and physicians often have difficulty identifying delirium, which leads to the unreasonable management of ICU patients [6]. Therefore, delirium generally results in poor outcomes in ICU patients, including prolonged duration of mechanical ventilation, increased length of stay, higher mortality, and greater cost [7–9].

The administration of analgesic sedatives to long-term mechanically ventilated patients is an effective means of relieving anxiety and agitation [10]. Midazolam and propofol are generally used for sedation [11–13]. However, these sedatives exhibit some adverse effects when used alone for long-term sedation, such as withdrawal reactions, delayed extubation from drug accumulation, hypertriglyceridemia, respiratory depression, and circulative function depression [14, 15]. Delirium is probably associated with these adverse effects, and it is also a main effect in ICU patients [13]. Sequential sedative use may reduce these adverse effects and lower the risk of delirium [16].

The risk factors of delirium in ICU patients are different among studies but generally include older age, higher acute physiology and chronic health evaluation (APACHE) II score, and exposure to sedatives and analgesics. However, few current studies examined delirium in patients who received sequential sedation. Therefore, the present study investigated the factors of delirium in patients who received sequential sedation in the ICU.

## 2. Materials and Methods

### 2.1. Patients

In this planned study, we selected patients from a previous study (ClinicalTrials.gov Identifier NCT02528513) during December 2015 to January 2017 in medical and surgical ICU in the West China Hospital of Sichuan University, Sichuan, China. The ethics committee of West China Hospital of Sichuan University approved this study. All of the patients involved in the trial signed an informed consent form and consented to participate in the study as appropriate. All patients older than 18 years old and younger than 80 years old, who were expected to receive mechanical ventilation longer than 72 hours and accepted sedation therapy, were recruited on admission to the ICU. Patients were excluded if they had a history of sedatives allergy (propofol, midazolam, or dexmedetomidine) and significant neurological diseases that would confound the evaluation of delirium, chronic renal failure, severe organ dysfunction, history of alcoholism, and taking antianxiety drugs or hypnotics.

**Figure 1:**
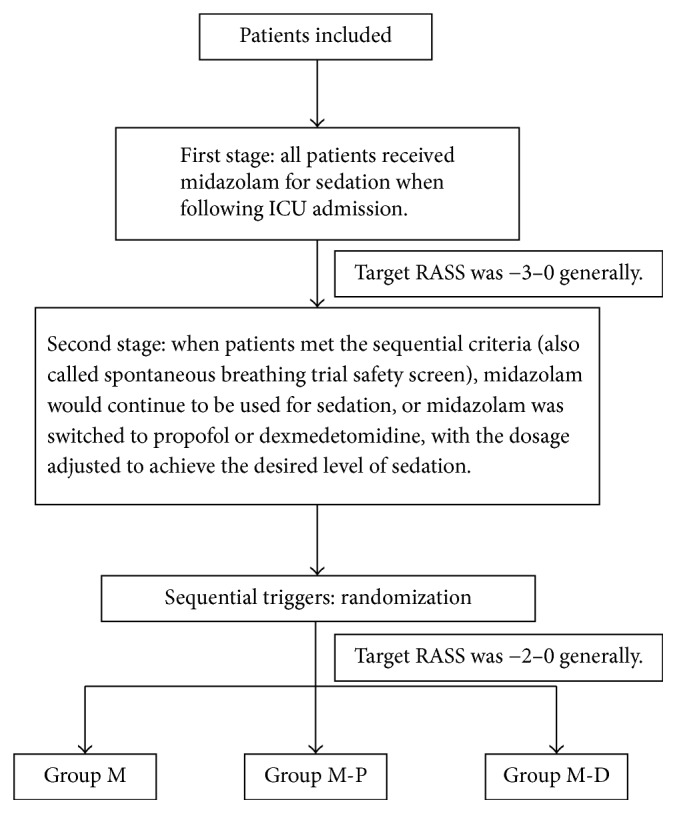
Sequential sedation process.

All patients in this study received continuous intravenous fentanyl for analgesia following ICU admission. Patient sedation treatment was divided into three groups (Figure 1): (1) the midazolam group (group M) throughout the sedative period until extubation; (2) the sequential use of midazolam and propofol group (group M-P), in which midazolam was switched to propofol when patients met sequential criteria; and (3) the sequential use of midazolam and dexmedetomidine group (group M-D), in which midazolam was switched to dexmedetomidine when patients met sequential criteria. The Richmond agitation-sedation scale (RASS) was used to assess the sedative level in each group [17]. Nurses continuously monitored sedation depth and adjusted the doses of sedative and analgesic drugs according to local sedation procedures to maintain the sedation level to an appropriate degree until patients successfully passed the spontaneous breathing trial (SBT) and were extubated [13, 18].

Sequential criteria are also called a spontaneous breathing trial safety screen. A patient's condition was severe following ICU admission. Patients were tested using the spontaneous breathing trial safety screen when their condition improved after a period of treatment. Enrolled patients passed the spontaneous breathing trial (SBT) safety screen if they exhibited adequate oxygenation (oxygen partial pressure ≥ 60 mmHg on a fraction of inspired oxygen ≤ 50% and a positive end-expiratory pressure ≤ 8 cmH_2_O), stable hemodynamics with no evidence of myocardial ischemia, and no significant use of vasopressors (dopamine or dobutamine ≤ 5 *μ*g/kg/min or norepinephrine ≤ 2 *μ*g/min) [19]. However, patients failed the SBT trial if they underwent a 30-minute SBT with 8 cmH_2_O pressure support ventilation, 5 cmH_2_O positive end-expiratory pressure, and 40% fraction of inspired oxygen [20].

### 2.2. Clinical Data

Patient demographic characteristics included age, gender, body mass index (BMI), allergic history, drinking and smoking status, and medical history, including the presence or absence of hypertension. APACHE II score, sequential organ failure assessment score (SOFA), and laboratory test results were determined within 24 hours of ICU admission. Other important information included the use of sedative and analgesic medications, mechanical ventilation status, and sequential sedation characteristics.

### 2.3. Delirium Assessment

The primary endpoint of this study was the occurrence of delirium. Patient evaluations were implemented using a CAM-ICU scale every 4 hours daily for a maximum of 28 days or until ICU discharge, whichever occurred first [8]. The CAM-ICU includes four parts: abrupt change in mental status, lack of attention, thinking disorder and change in consciousness level [1]. The research team performed delirium assessments daily and recorded the data synchronously.

### 2.4. Statistical Analysis

Statistical analyses were performed using SPSS 23.0 (Statistical Product and Service Solutions, IBM, USA). Continuous variables are presented as medians and interquartile ranges according to their distribution. Categorical variables are presented as percentages. The Mann–Whitney *U* test was used to compare differences in continuous variables between delirium and no delirium groups. Chi-square and Fisher's exact probability tests were used to compare differences in categorical variables between two groups. Risk factors were analyzed using Cox proportional hazards regression. Any variables that exhibited *p* < 0.2 after univariate Cox proportional regression analysis or potential variables associated with delirium were included in the multivariate Cox proportional regression analysis. The cut-off point of age, APACHE II score, and SOFA score were determined using interquartiles to achieve the best discrimination between groups with or without delirium. *p* < 0.05 was considered statistically significant.

The sample size for this study was estimated according to the incidence of delirium from previous studies that revealed a historical ICU delirium incidence between 30% and 53.8% [21, 22]. The sample size limited the number of variables in the multivariate regression model, following the generally accepted rule of one variable per ten patients [23]. We estimated that the final multivariate regression model would include approximately 5 variables. Therefore, we estimated a delirium group minimum sample size of 50 patients. We finally estimated that at least 100 patients should be included in this study based on historical delirium incidence and the delirium group minimum sample size.

## 3. Results

**Figure 2:**
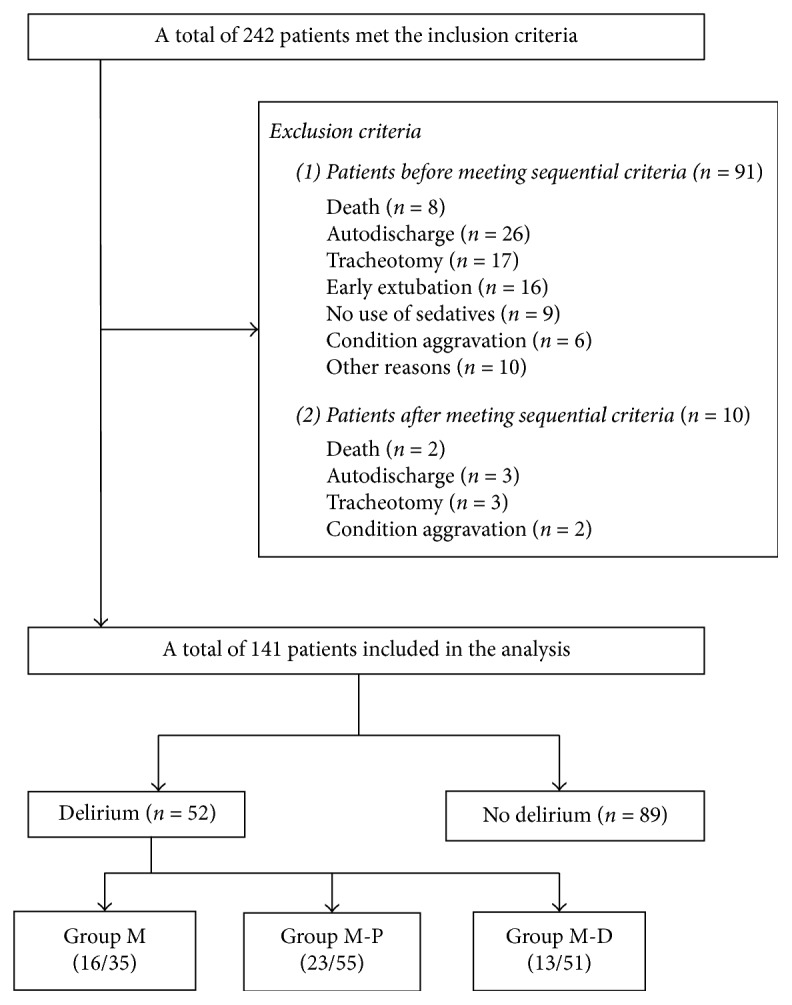
Study flow chart.

A total of 242 met our inclusion criteria. Ninety-one patients were excluded before meeting the sequential criteria, including 8 patients for death, 26 patients for autodischarge, 17 patients for tracheotomy, 16 patients for early extubation, 9 patients for no use of sedatives, 6 patients for condition aggravation, and another 10 patients for other reasons. The remaining 10 patients met the sequential criteria and received assigned sedatives, but these patients were excluded for death, tracheotomy, autodischarge, and condition aggravation. A total of 141 patients were recruited for the analysis: 35 patients in group M, 55 patients in group M-P, and 51 patients in group M-D.  Figure 2 presents the study flow. There were 52 patients diagnosed with delirium in total. Sixteen patients developed delirium in group M (16/35), and 23 patients developed delirium in group M-P (23/55), and 13 patients developed delirium in group M-D (13/51).

Table 1 presents demographic data and baseline characteristics of patients with or without delirium. Delirious patients were significantly older (54 (47–65) versus 49 (38–61), *p* = 0.023), exhibited higher SOFA scores at ICU admission (≥14) (34.6% versus 19.1%, *p* = 0.040), and have higher maintenance dose of midazolam (mg/kg/d) (1.896 (1.440–2.424) versus 1.440 (0.960–1.992), *p* = 0.001) and a higher maintenance dose of fentanyl when patients met sequential criteria (*μ*g/kg/d) (19.32 (14.40–24.24) versus 18.24 (14.40–21.12), *p* = 0.035).

Univariate Cox proportional regression (Table 2) revealed that age ≥ 51, regular smoking, SOFA score ≥ 14, the first dose of fentanyl at ICU admission, and maintenance dose of midazolam and fentanyl increased the risk of developing delirium, whereas sequential sedation using different sedatives may reduce delirium risk. Multivariate Cox proportional regression analysis (Table 3) revealed that older age (≥51) (RR = 2.432, 95% CL 1.316–4.494, *p* = 0.005), higher SOFA score (≥14) (RR = 2.022, 95% CL 1.076–3.798, *p* = 0.029), regular smoking (RR = 2.366, 95% CL 1.277–4.382, *p* = 0.006), higher maintenance dose of midazolam (RR = 1.052, 95% CL 1.000–1.107, *p* = 0.049), and higher maintenance dose of fentanyl (RR = 1.045, 95% CL 1.019–1.072, *p* = 0.001) were independent risk factors of developing delirium in patients with sequential sedation. Sequential sedation with dexmedetomidine (RR = 0.448, 95% CL 0.209–0.963, *p* = 0.040) was associated with lower risk of delirium compared to midazolam.

## 4. Discussion

ICU patients exhibit a high incidence of the occurrence of delirium [22, 24, 25]. The range of reported delirium incidences is wide, which is likely due to the use of different patients, objectives, and designs in studies. The total incidence of delirium was 37% in the present study. This incidence is generally consistent with the incidences reported in previous studies [22, 24, 26].

Many risk factors of delirium were identified in numerous relevant studies, such as older age, hypertension, respiratory disorder, alcohol abuse, smoking, greater illness severity, dementia, and medications [6, 27]. Our study bolstered these opinions about the risk factors of delirium. However, our study was the first to demonstrate how sequential sedation management affected the development of delirium and to identify the risk factors associated with delirium.

Age is the most basic characteristic for patients, but older age is a predisposing risk factor for ICU delirium [28–30]. The present study found that patients older than 51 were at almost 2.4 times (RR = 2.432, *p* = 0.005) the risk for development of delirium than patients younger than 51. This difference means that delirium developed easily in older ICU patients who received sedation therapy. Some patient's living habits cannot also be ignored, such as alcohol abuse and regular smoking. Alcohol abuse and smoking are well-known risk factors for ICU delirium [28, 31]. Our study did not find that history of alcohol abuse affected the development of delirium. However, patients with regular smoking had a higher risk for developing delirium (RR = 2.366, *p* = 0.006).

Previous studies demonstrated that the development of delirium was associated with features of the acute illness, such as categories of acute illness and the disease severity of individual patients [21, 31, 32]. APACHE II and SOFA scores are two valid tools to evaluate the disease severity in the ICU [33, 34]. We did not find an association between delirium and APACHE II score in the final multivariate regression model. However, admission SOFA score was a significantly independent predictor for the development of delirium in multivariate regression analysis. We found that the prevalence of a SOFA score ≥ 14 was significantly higher in patients with delirium than patients without delirium (34.6% versus 19.1%, *p* = 0.040).

**Figure 3:**
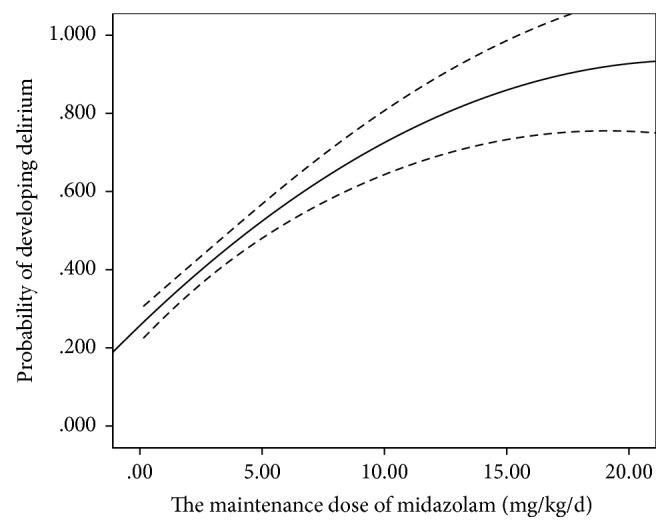
The maintenance dose of midazolam when patients met the sequential criteria and the probability of developing delirium. The probability of delirium increased with the maintenance dose of midazolam.

**Figure 4:**
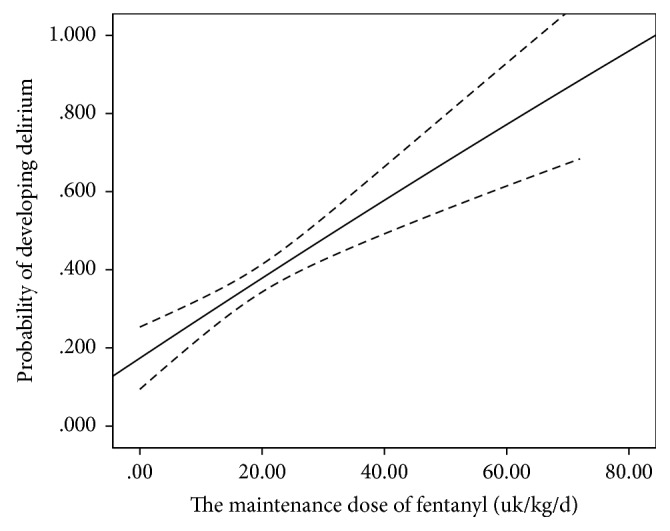
The maintenance dose of fentanyl when patients met the sequential criteria and the probability of developing delirium. The probability of delirium also increased with the maintenance dose of fentanyl.

Sedation and analgesia management is often required in ICU patients to relieve anxiety, pain, or other physical discomfort [15]. However, benzodiazepine use is generally related to prolonged durations of MV and the length of ICU stay [35]. Benzodiazepines are also associated with sleep disturbance, posttraumatic stress disorder, and depression in ICU patients [36, 37]. These factors may lead to the development of delirium in ICU patients [28]. Higher doses of benzodiazepines and fentanyl increased the risk of delirium in burn and trauma ICU patients [38, 39]. Researchers found that benzodiazepine use in the cardiovascular ICU also increased the risk of delirium [24]. Our results demonstrated that benzodiazepine and fentanyl use was reliably related to the development of delirium. We further found that the maintenance doses of midazolam and fentanyl were associated with the development of delirium when patients met the sequential criteria. Patients were at increased risk of delirium when exposed to higher midazolam maintenance (RR = 1.052, *p* = 0.049) (Figure 3). Similarly, higher fentanyl maintenance also increased the risk of developing delirium (RR = 1.045, *p* = 0.001) (Figure 4). Therefore, maintenance doses of midazolam and fentanyl may be reduced appropriately before patients satisfy the sequential criteria and sequentially receive another sedative when their condition has improved. However, benzodiazepines may be used in larger doses for alcohol withdrawal if patients had a serious history of alcohol abuse [6].

**Figure 5:**
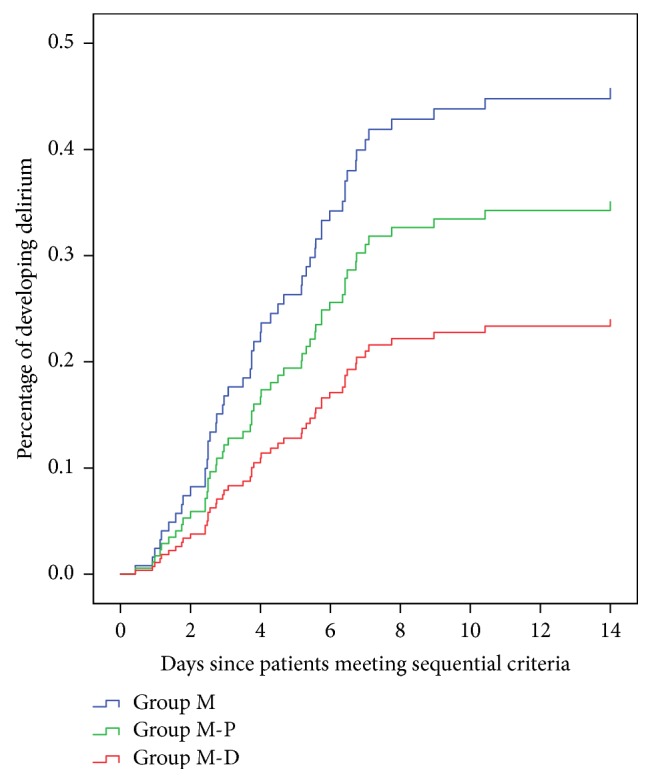
The percentage of developing delirium for sequential sedation patients in group M, group M-P, and group M-D (adjusted other variables in multivariate Cox proportional regression).

Nonpharmacological therapies and pharmacological management are used to prevent the development of delirium to protect against the adverse effects of delirium. Alpha-2 agonists (dexmedetomidine) decrease the incidence of delirium [40–42]. This study used dexmedetomidine for sequential sedation, except when midazolam and propofol were used. Multivariate regression analysis found that sequential sedation with dexmedetomidine was an actually protective factor against delirium (RR = 0.448, *p* = 0.040), and the percentage of developing delirium was lowest in group M-D (Figure 5). This result demonstrates that patients benefit from the sequential use of midazolam and dexmedetomidine, and it reduces the risk for developing delirium. The pharmacology of dexmedetomidine is different from that of benzodiazepines and propofol. It is an *α*2-receptor agonist that provides antianxiety and analgesia by acting on the nucleus coeruleus [43]. Patients who receive dexmedetomidine generally exhibit light sedation and are more cooperative, communicative, and arousable [44]. Sedation with dexmedetomidine improves sleep quality and decreases the incidence of complications [42]. The above-mentioned characteristics are likely associated with delirium prevention. The use of benzodiazepines and propofol alone for long-term sedation is associated with some adverse effects [13, 16], and these effects may lead to the development of delirium. Sequential use may reduce these adverse effects. This study examined the characteristics of sequential sedation and pharmacology of dexmedetomidine and found that the sequential use of midazolam and dexmedetomidine for long-term sedation was an effective and safe strategy to prevent delirium in ICU patients.

To the best of our knowledge, this report is the first study to evaluate the potential risk factors for the development of delirium in patients who receive sequential sedation. However, our study has some limitations, including small sample size and potential biases inherent to research. More similar studies are needed to confirm our results.

## 5. Conclusions

The present study demonstrated that older age (≥51), regular smoking, higher SOFA score (≥14), and increased maintenance of midazolam and fentanyl when patients met sequential criteria were significant risk factors of delirium in patients who received sequential sedation. The results of this study also demonstrated that sequential sedation with dexmedetomidine was a protective method to prevent delirium. Our results suggest that management strategies may sometimes require alteration to reduce the incidence and severity of delirium in sequential sedation patients.

## Figures and Tables

**Table 1:** Characteristics of patients with and without delirium in this study.

Variables	Delirium (*n* = 52)	Nondelirium (*n* = 89)	*p*
*Baseline characteristics of patients*			
Age (years)	54 (47–65)	49 (38–61)	0.023^*※※*^
<51	19 (36.5)	51 (57.3)	0.017^*※※*^
≥51	33 (63.5)	38 (42.7)	
Male (*n*, %)	38 (73.1)	56 (62.9)	0.217
Body mass index (BMI)	23.35 (20.24–26.70)	23.77 (21.86–25.79)	0.632
Allergic history (*n*, %)			0.358
Yes	3 (5.8)	2 (2.2)	
No	49 (94.2)	87 (97.8)	
Regular drinking (*n*, %)			0.838
Yes	19 (36.5)	31 (34.8)	
No	33 (63.5)	58 (65.2)	
Regular smoking (*n*, %)			0.175
Yes	21 (40.4)	26 (29.2)	
No	31 (59.6)	63 (73.8)	
Hypertension (*n*, %)			0.525
Yes	11 (21.2)	15 (16.9)	
No	41 (78.8)	74 (83.1)	
ICU admission diagnosis (*n*, %)			0.240
Pneumonia	13 (25.0)	22 (24.7)	
Sepsis	11 (21.2)	20 (22.5)	
Trauma	15 (28.8)	15 (16.9)	
Pancreatitis	13 (25.0)	27 (30.3)	
other digestive disease	0 (0.0)	5 (5.6)	
*(1) Patients at ICU admission (before meeting the sequential criteria)*			
APACHE II	19 (14–25)	19 (14–23)	0.771
<23	38 (73.1)	68 (76.4)	0.659
≥23	14 (26.9)	21 (23.6)	
SOFA score	10 (8–14)	10 (7–13)	0.595
<14	34 (65.4)	72 (80.9)	0.040^*※※*^
≥14	18 (34.6)	17 (19.1)	
Cholesterol (mmol/L)	2.31 (1.86–3.28)	2.29 (1.44–3.07)	0.082
Triglyceride (mmol/L)	1.41 (0.79–2.43)	1.40 (0.82–2.21)	0.584
The first dose of midazolam (mg/kg/d)	1.728 (1.380–2.184)	1.680 (1.320–1.920)	0.145
The first dose of fentanyl (*μ*g/kg/d)	18.48 (15.36–23.04)	17.28 (14.40–19.92)	0.068
*(2) After meeting the sequential criteria*			
The maintenance dose of midazolam (mg/kg/d)	1.896 (1.440–2.424)	1.440 (0.960–1.992)	0.001^*※※*^
The maintenance dose of fentanyl (*μ*g/kg/d)	19.32 (14.40–24.24)	18.24 (14.40–21.12)	0.035^*※※*^
The accumulated dose of midazolam (mg)	336 (200–601)	400 (200–750)	0.603
The accumulated dose of fentanyl (mg)	4.315 (2.5–7.5)	4.250 (3.0–7.0)	0.710
Blood glucose (mmol/L)	7.515 (5.930–11.035)	8.575 (6.870–10.215)	0.257
Triglyceride (mmol/L)	1.445 (1.020–2.400)	1.860 (1.245–2.415)	0.201
*(3) Continuous use of midazolam alone or sequential use of midazolam and propofol/dexmedetomidine after meeting the sequential criteria*			
Time of meeting the sequential criteria to stop sedation (hours)	33.00 (20.75–56.75)	25.00 (19.75–47.25)	0.243
Time of meeting the sequential criteria for extubation (hours)	48.75 (24.85–70.0)	34.75 (24.10–50.25)	0.317
Time of MV (hours)	144.75 (109.00–200.25)	160.00 (109.50–209.50)	0.346
ICU length of stay (days)	12.91 (9.89–18.89)	14.85 (9.90–19.98)	0.462
Hospital length of stay (days)	22.33 (15.32–40.06)	19.92 (14.85–30.52)	0.357
NIPPV after extubation (*n*, %)			0.481
Yes	14 (26.9)	29 (32.6)	
No	38 (73.1)	60 (67.4)	
Vasoactive agent (*n*, %)			0.417
Yes	13 (26.0)	29 (32.6)	
No	37 (74.0)	60 (67.4)	
Sequential sedatives (*n*, %)			0.101
Midazolam (group M-M)	16 (30.8)	19 (21.3)	
Propofol (group M-P)	23 (44.2)	32 (36.0)	
Dexmedetomidine (group M-D)	13 (25.0)	38 (42.7)	

BMI, body mass index; APACHE, acute physiology and chronic health evaluation; SOFA, sequential organ failure assessment; MV, mechanical ventilation; NIPPV, noninvasive positive pressure ventilation; ^*※※*^*p* < 0.05.

**Table 2:** Univariate Cox proportional regression of ICU delirium in sequential sedation patients.

Variables	RR	95% CL	*p*
Age (≥51)	1.957	1.112–3.444	0.020^*※※*^
Allergic history	2.013	0.626–6.467	0.240
Regular drinking	1.078	0.613–1.895	0.795
Regular smoking	1.580	0.908–2.750	0.106^*※*^
Hypertension	1.249	0.642–2.430	0.513
APACHE II (≥23)^a^	1.146	0.621–2.115	0.663
SOFA score (≥14)^a^	1.801	1.016–3.190	0.044^*※※*^
The first dose of midazolam (mg/kg/d)^a^	1.095	0.833–1.438	0.515
The first dose of fentanyl (*μ*g/kg/d)^a^	1.040	1.004–1.077	0.030^*※※*^
The maintenance dose of midazolam (mg/kg/d)^b^	1.048	1.001–1.097	0.043^*※※*^
The maintenance dose of fentanyl (*μ*g/kg/d)^b^	1.025	1.004–1.047	0.021^*※※*^
The accumulated dose of midazolam (mg)^b^	1.000	0.999–1.001	0.960
The accumulated dose of fentanyl (mg)^b^	0.968	0.916–1.023	0.252
Time of patients meeting sequential criteria to stop sedation^c^	1.003	0.995–1.011	0.486
Time of patients meeting sequential criteria for extubation^c^	1.002	0.995–1.009	0.635
Sequential sedatives^c^			
Midazolam (group M-M)	1.000	Ref.	
Propofol (group M-P)	0.863	0.456–1.634	0.652
Dexmedetomidine (group M-D)	0.451	0.217–0.939	0.033^*※※*^

^a^Patients at ICU admission (before meeting the sequential criteria). ^b^When patients met the sequential criteria. ^c^After patients met the sequential criteria for extubation. ^*※*^*p* < 0.2, ^*※※*^*p* < 0.05.

**Table 3:** Multivariate Cox proportional regression of ICU delirium in sequential sedation patients.

Risk factors	RR	95% CL	*p*
Age (≥51)	2.432	1.316–4.494	0.005^*※※*^
Regular smoking	2.366	1.277–4.382	0.006^*※※*^
SOFA score (≥14)^a^	2.022	1.076–3.798	0.029^*※※*^
The first dose of fentanyl (*μ*g/kg/d)^a^	0.992	0.951–1.035	0.772
The maintenance dose of midazolam (mg/kg/d)^b^	1.052	1.000–1.107	0.049^*※※*^
The maintenance dose of fentanyl (*μ*g/kg/d)^b^	1.045	1.019–1.072	0.001^*※※*^
Sequential sedatives^c^			
Midazolam (group M-M)	1.000	Ref.	
Propofol (group M-P)	0.706	0.364–1.369	0.303
Dexmedetomidine (group M-D)	0.448	0.209–0.963	0.040^*※※*^

^a^Patients at ICU admission (before meeting the sequential criteria). ^b^When patients met the sequential criteria. ^c^After patients met the sequential criteria for extubation. ^*※※*^*p* < 0.05.

## List of Abbreviations

MV: Mechanical ventilation
ICU: Intensive care unit
CAM-ICU: Confusion Assessment Method for the Intensive Care Unit
SBT: Spontaneous breathing trial
BMI: Body mass index
APACHE: Acute physiology and chronic health evaluation
RASS: Richmond agitation-sedation scale
SOFA: Sequential organ failure assessment
NIPPV: Noninvasive positive pressure ventilation.
